# A new species of the horned toad *Megophrys* Kuhl & Van Hasselt, 1822 (Anura, Megophryidae) from southwest China

**DOI:** 10.3897/zookeys.943.50343

**Published:** 2020-06-22

**Authors:** Ning Xu, Shi-Ze Li, Jing Liu, Gang Wei, Bin Wang

**Affiliations:** 1 Biodiversity Conservation Key Laboratory, Guiyang College, Guiyang, 550002, China Guiyang College Guiyang China; 2 CAS Key Laboratory of Mountain Ecological Restoration and Bioresource Utilization & Ecological Restoration Biodiversity Conservation Key Laboratory of Sichuan Province, Chengdu Institute of Biology, Chinese Academy of Sciences, Chengdu 610041, China Moutai Institute Renhuai China; 3 Department of Food Science and Engineering, Moutai Institute, Renhuai 564500, China Chengdu Institute of Biology Chengdu China

**Keywords:** Taxonomy, molecular phylogenetic analysis, morphology

## Abstract

A new species of the genus *Megophrys* is described from Guizhou Province, China. Molecular phylogenetic analyses based on mitochondrial DNA and nuclear DNA sequences all strongly supported the new species as an independent clade sister to *M. minor* and *M. jiangi*. The new species could be distinguished from its congeners by a combination of the following characters: body size moderate (SVL 43.4–44.1 mm in males, and 44.8–49.8 mm in females; vomerine teeth absent; tongue not notched behind; a small horn-like tubercle at the edge of each upper eyelid; tympanum distinctly visible, rounded; two metacarpal tubercles on palm; relative finger lengths II < I < V < III; toes without webbing; heels overlapping when thighs are positioned at right angles to the body; tibiotarsal articulation reaching the level between tympanum and eye when leg stretched forward; in breeding males, an internal single subgular vocal sac in male, and the nuptial pads with black spines on dorsal surface of bases of the first two fingers.

## Introduction

The Asian horned toad *Megophrys* Kuhl & Van Hasselt, 1822 (Anura: Megophryidae Bonaparte, 1850) is widely distributed in eastern and central China, throughout southeastern Asia, and extending to the islands of the Sunda Shelf and the Philippines (Frost 2020). The taxonomic arrangements especially on generic assignments of the group have been controversial for a long time (e.g., Tian and Hu 1983; Dubois 1987; Lathrop 1997; Rao and Yang 1997; Jiang et al. 2003; Delorme et al. 2006; Fei et al. 2009; Chen et al. 2016; Fei and Ye 2016; Deuti et al. 2017; Mahony et al. 2017; Frost 2019). Nevertheless, all molecular phylogenetic studies revealed this group as a monophyletic group which corresponds to the family (Chen et al. 2016; Mahony et al. 2017; Liu et al. 2018; Li et al. 2018; Liu et al. 2020; Wang et al. 2020), and thus many researchers considered it as a large genus *Megophryssensu lato* (Mahony et al. 2017; Li et al. 2018; Liu et al. 2018, 2020; Frost 2020; Wang et al. 2020) although several studies divided the taxa of the group into different genera and subgenera, thus introducing better resolution of relationships within the family (Chen et al. 2016; Fei and Ye 2016; Deuti et al. 2017; Liu et al. 2018).

The large genus *Megophrys* currently contains 98 species, of which 41 species were described in the last decade (Frost 2020; Liu et al. 2020). Many cryptic species in the genus are indicated by molecular phylogenetic analyses (Chen et al. 2016; Liu et al. 2018) of which several have been described recently (e.g., Wang et al. 2019; Liu et al. 2020). Obviously, more cryptic species need to be verified and described in detail.

During field surveys in the Chishui National Nature Reserve, Chishui City, Guizhou Province, China, we collected a series of *Megophrys* specimens. Our molecular phylogenetic analyses and morphological comparisons support it as an undescribed species, and it is described herein as a new species.

## Materials and methods

### Sampling

**Figure 1.**
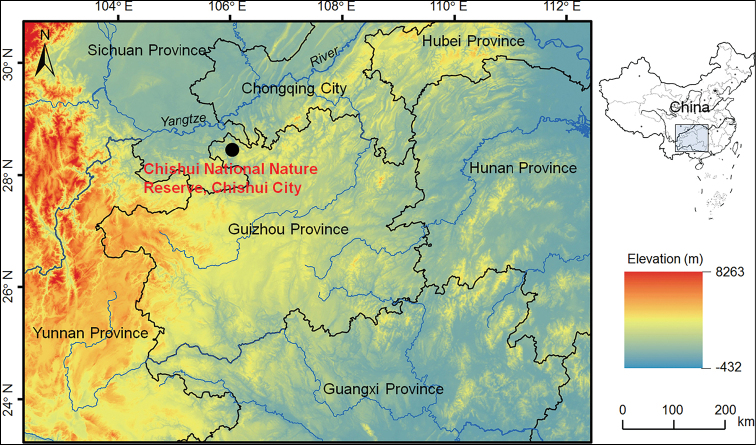
Geographical location of the type locality, Chishui National Nature Reserve, Chishui City, Guizhou Province, China, of *Megophrys chishuiensis* sp. nov.

Three adult males and five adult females of the undescribed species were collected in Chishui National Nature Reserve, Chishui City, Guizhou Province, China (Suppl. material 1: Table S1; Fig. 1). In the field, the toads were euthanized using isoflurane, and the specimens were fixed in 75% ethanol. Tissue samples were taken and preserved separately in 99% ethanol prior to fixation. The specimens were deposited in Chengdu Institute of Biology, Chinese Academy of Sciences (**CIB, CAS**).

### Molecular data and phylogenetic analyses

Six specimens of the undescribed species were included in the molecular analyses (Suppl. material 2: Table S2). Total DNA was extracted using a standard phenol-chloroform extraction protocol (Sambrook et al. 1989). Two fragments of the mitochondrial genes encoding16S rRNA and cytochrome oxidase subunit I (COI) were amplified using the primers in Simon et al. (1994) and Che et al. (2012), respectively. PCR were under the following conditions: 37 cycles at 94 °C for 4 min, 95 °C for 1 min, 53 °C (for 16S rRNA)/47 °C (for COI) for 30 sec, and 72 °C for 1 min followed by a 8-min extension at 72 °C. The nuclear gene sequences encoding brain-derived neurotrophic factor (BDNF) and recombination activating gene 1 (RAG1) were amplified using the primers and protocols in Vieites et al. (2007) and Shen et al. (2013), respectively (Suppl. material 3: Table S3). All PCR products were purified with spin columns, and then were sequenced with primers same as used in PCR. Sequencing was conducted using an ABI3730 automated DNA sequencer in Shanghai DNA BioTechnologies Co., Ltd. (Shanghai, China). All sequences were deposited in GenBank (for accession numbers see Suppl. material 2: Table S2).

For molecular analyses, the available sequence data for congeners of *Megophrys* were downloaded from GenBank (Suppl. material 2: Table S2), primarily from previous studies (Chen et al. 2017; Liu et al. 2018). For phylogenetic analyses, corresponding sequences of one *Leptobrachella oshanensis* (Liu, 1950) and one *Leptobrachium boringii* (Liu, 1945) were also downloaded (Suppl. material 2: Table S2), and used as outgroups according to Mahony et al. (2017). Sequences were assembled and aligned in BioEdit v. 7.0.9.0 (Hall 1999) with default settings. Alignments were checked by eye and revised manually if necessary. To avoid bias in alignments, GBLOCKS v. 0.91.b (Castresana 2000) with default settings was used to extract regions of defined sequence conservation from the length-variable 16S gene fragments. Non-sequenced fragments were defined as missing loci. For phylogenetic analyses, two datasets were obtained, i.e., two-mitochondrial genes concatenated dataset of 16S+COI and two-nuclear genes concatenated dataset of RAG1+BDNF.

**Table 1.** Measurements of the adult specimens of *Megophrys chishuiensis* sp. nov. Units are given in mm. See abbreviations for the morphological characters in Materials and methods section.

	Male (*N* = 3)		Female (*N* = 5)	
	Range	Mean ± SD	Range	Mean ± SD
SVL	43.4–44.1	43.6 ± 0.4	44.8–49.8	47.8 ± 2.0
HDL	11.4–11.9	11. 7 ± 0.3	11.2–12.7	11.7 ± 0.6
HDW	13.0–13.9	13.5 ± 0.5	13.8–15.4	14.7 ± 0.6
SL	4.2–5.3	4.8 ± 0.6	4.3–5.4	4.9 ± 0.4
IND	5.0–5.2	5.1 ± 0.1	4.5–5.8	5.1 ± 0.6
IOD	3.1–3.5	3.3 ± 0.2	3.1–4.3	3.5 ± 0.5
ED	4.4–5.0	4.7 ± 0.3	4.9–5.7	5.4 ± 0.3
UEW	4.1–4.9	4.4 ± 0.4	4.1–5.2	4.7 ± 0.4
TYD	2.8–3.5	3.2 ± 0.4	2.2–3.1	2.7 ± 0.3
LAL	18.4–20.0	19.0 ± 0.9	20.3–22.0	21.3 ± 0.7
LW	4.5–4.7	4.6 ± 0.1	3.2–3.6	3.4 ± 0.2
HLL	59.4–65.1	63.0 ± 3.1	64.2–75.6	70.7 ± 4.1
THL	17.2–21.3	19.8 ± 2.2	20.4–23.8	22.1 ± 1.3
TL	18.0–21.7	20.1 ± 1.9	22.0–24.0	23.2 ± 0.8
TW	4.6–5.1	4.9 ± 0.3	5.0–5.8	5.3 ± 0.3
TFL	28.0–30.2	28.9 ± 1.2	30.1–33.0	31.3 ± 1.1
FL	18.5–19.2	18.9 ± 2.3	18.8–22.1	21.0 ± 1.4

Phylogenetic relationships were reconstructed based on the mitochondrial DNA data and nuclear DNA data, respectively. Phylogenetic analyses were conducted using maximum likelihood (ML) and Bayesian Inference (BI) methods, implemented in PhyML v. 3.0 (Guindon et al. 2010) and MrBayes v. 3.12 (Ronquist and Huelsenbeck 2003), respectively. To avoid under- or over-parameterization (Lemmon and Moriarty 2004; McGuire et al. 2007), the best partition scheme and the best evolutionary model for each partition were chosen for the phylogenetic analyses using PARTITIONFINDER v. 1.1.1 (Robert et al. 2012). In the analyses, 16S, each codon position of the protein-coding genes (COI, RAG1 and BDNF) were defined, and Bayesian Inference Criteria (BIC) was used. As a result, the analyses selected the best partition scheme (i.e., 16S gene/each codon position of COI gene) and the GTR+ G + I model for each partition for mitochondrial DNA dataset, and as well, selected the best partition scheme (i.e., each codon position of RAG1 and BDNF genes) and the GTR+ G + I as the best model for all codon position of RAG1 and BDNF genes. For the ML tree, branch supports were drawn from 10000 non-parametric bootstrap replicates. In BI analyses, two runs each with four Markov chains were run for 40 million generations with sampling every 1000 generations. The first 25% of generations were removed as the “burn-in” stage followed by calculation of Bayesian posterior probabilities and the 50% majority-rule consensus of the post burn-in trees sampled at stationarity. Finally, genetic distance between species under uncorrected *p*-distance model was estimated on 16S gene sequences using MEGA v. 6.06 (Tamura et al. 2011).

### Morphological comparisons

All adult specimens of the undescribed species were measured. The terminology and methods followed Fei et al. (2009). Measurements were taken with a dial caliper to 0.1 mm. Seventeen morphometric characters of adult specimens were measured:

**ED** eye diameter (distance from the anterior corner to the posterior corner of the eye);

**FL** foot length (distance from tarsus to the tip of fourth toe);

**HDL** head length (distance from the tip of the snout to the articulation of jaw);

**HDW** maximum head width (greatest width between the left and right articulations of jaw);

**HLL** hindlimb length (maximum length from the vent to the distal tip of the Toe IV);

**IND** internasal distance (minimum distance between the inner margins of the external nares);

**IOD** interorbital distance (minimum distance between the inner edges of the upper eyelids);

**LAL** length of lower arm and hand (distance from the elbow to the distal end of the Finger IV);

**LW** lower arm width (maximum width of the lower arm);

**SL** snout length (distance from the tip of the snout to the anterior corner of the eye);

**SVL** snout-vent length (distance from the tip of the snout to the posterior edge of the vent);

**TFL** length of foot and tarsus (distance from the tibiotarsal articulation to the distal end of the Toe IV);

**THL** thigh length (distance from vent to knee);

**TL** tibia length (distance from knee to tarsus);

**TYD** maximal tympanum diameter;

**TW** maximal tibia width;

**UEW** upper eyelid width (greatest width of the upper eyelid margins measured perpendicular to the anterior-posterior axis).

We compared morphological characters of the undescribed species with *Megophrys* congeners. Comparative data were obtained from related species as described in literature (Table 2).

**Table 2.** References for morphological characters for congeners of the genus *Megophrys*.

Species	Literature
*M. aceras* Boulenger, 1903	Taylor 1962
*M. acuta* Wang, Li & Jin, 2014	Li et al. 2014
*M. ancrae* Mahony, Teeling & Biju, 2013	Mahony et al. 2013
*M. angka* Wu, Suwannapoom, Poyarkov, Chen, Pawangkhanant, Xu, Jin, Murphy & Che, 2019	Wu et al. 2019
*M. auralensis* Ohler, Swan & Daltry, 2002	Ohler et al. 2002
*M. baluensis* (Boulenger, 1899)	Boulenger 1899
*M. baolongensis* Ye, Fei & Xie, 2007	Ye et al. 2007
*M. binchuanensis* Ye & Fei, 1995	Ye and Fei 1995
*M. binlingensis* Jiang, Fei & Ye, 2009	Fei et al. 2009
*M. boettgeri* (Boulenger, 1899)	Fei et al. 2012
*M. brachykolos* Inger & Romer, 1961	Inger and Romer 1961
*M. carinense* (Boulenger, 1889)	Fei et al. 2009
*M. caobangensis* Nguyen, Pham, Nguyen, Luong & Ziegler, 2020	Nguyen et al. 2020
*M. caudoprocta* Shen, 1994	Fei et al. 2012
*M. cheni* (Wang & Liu, 2014)	Wang et al. 2014
*M. chuannanensis* (Fei, Ye & Huang, 2001)	Fei et al. 2012
*M. damrei* Mahony, 2011	Mahony 2011
*M. daweimontis* Rao & Yang, 1997	Fei et al. 2012
*M. dongguanensis* Wang & Wang, 2019	Wang et al. 2019
*M. dringi* Inger, Stuebing & Tan, 1995	Inger et al. 1995
*M. edwardinae* Inger, 1989	Inger 1989
*M. elfina* Poyarkov, Duong, Orlov, Gogoleva, Vassilieva, Nguyen, Nguyen, Nguyen, Che & Mahony, 2017	Poyarkov et al. 2017
*M. fansipanensis* Tapley, Cutajar, Mahony, Nguyen, Dau, Luong, Le, Nguyen, Nguyen, Portway, Luong & Rowley, 2018	Tapley et al. 2018
*M. feae* Boulenger, 1887	Fei et al. 2009
*M. feii* Yang, Wang & Wang, 2018	Yang et al. 2018
*M. flavipunctata* Mahony, Kamei, Teeling & Biju, 2018	Mahony et al. 2018
*M. gerti* (Ohler, 2003)	Ohler 2003
*M. gigantica* Liu, Hu & Yang, 1960	Fei et al. 2012
*M. glandulosa* Fei, Ye & Huang, 1990	Fei et al. 2012
*M. hansi* (Ohler, 2003)	Ohler 2003
*M. himalayana* Mahony, Kamei, Teeling & Biju, 2018	Mahony et al. 2018
*M. hoanglienensis* Tapley, Cutajar, Mahony, Nguyen, Dau, Luong, Le, Nguyen, Nguyen, Portway, Luong & Rowley, 2018	Tapley et al. 2018
*M. huangshanensis* Fei & Ye, 2005	Fei et al. 2012
*M. insularis* (Wang, Liu, Lyu, Zeng & Wang, 2017)	Wang et al. 2017a
*M. intermedia* Smith, 1921	Rao and Yang 1997
*M. jiangi* Liu, Li, Wei, Xu, Cheng, Wang & Wu, 2020	Liu et al. 2020
*M. jingdongensis* Fei & Ye, 1983	Fei et al. 2012
*M. jinggangensis* (Wang, 2012)	Wang et al. 2012
*M. jiulianensis* Wang, Zeng, Lyu & Wang, 2019	Wang et al. 2019
*M. kalimantanensis* Munir, Hamidy, Matsui, Iskandar, Sidik & Shimada, 2019	Munir et al. 2019
*M. kobayashii* Malkmus & Matsui, 1997	Malkmus and Matsui 1997
*M. koui* Mahony, Foley, Biju & Teeling, 2017	Mahony et al. 2017
*M. kuatunensis* Pope, 1929	Fei et al. 2012
*M. lancip* Munir, Hamidy, Farajallah & Smith, 2018	Munir et al. 2018
*M. leishanensis* Li, Xu, Liu, Jiang, Wei & Wang, 2018	Li et al. 2018
*M. lekaguli* Stuart, Chuaynkern, Chan-ard & Inger, 2006	Stuart et al. 2006
*M. liboensis* (Zhang, Li, Xiao, Li, Pan, Wang, Zhang & Zhou, 2017)	Zhang et al. 2017
*M. ligayae* Taylor, 1920	Taylor 1920
*M. lini* (Wang & Yang, 2014)	Wang et al. 2014
*M. lishuiensis* (Wang, Liu & Jiang, 2017)	Wang et al. 2017b
*M. longipes* Boulenger, 1886	Taylor 1962
*M. major* Boulenger, 1908	Mahony et al. 2018
*M. mangshanensis* Fei & Ye, 1990	Fei et al. 2012
*M. maosonensis* Bourret, 1937	Bourret 1937
*M. medogensis* Fei, Ye & Huang, 1983	Fei et al. 2012
*M. megacephala* Mahony, Sengupta, Kamei & Biju, 2011	Mahony et al. 2011
*M. microstoma* (Boulenger, 1903)	Fei et al. 2012
*M. minor* Stejneger, 1926	Fei et al. 2012
*M. montana* Kuhl & Van Hasselt, 1822	Kuhl and Van Hasselt 1822
*M. monticola* (Günther, 1864)	Mahony et al. 2018
*M. mufumontana* Wang, Lyu & Wang, 2019	Wang et al. 2019
*M. nankiangensis* Liu & Hu, 1966	Fei et al. 2012
*M. nankunensis* Wang, Zeng & Wang, 2019	Wang et al. 2019
*M. nanlingensis* Lyu, Wang, Liu & Wang, 2019	Wang et al. 2019
*M. nasuta* (Schlegel, 1858)	Taylor 1962
*M. obesa* Wang, Li & Zhao, 2014	Wang et al. 2014
*M. ombrophila* Messenger & Dahn, 2019	Munir et al. 2019
*M. omeimontis* Liu, 1950	Fei et al. 2009
*M. oreocrypta* Mahony, Kamei, Teeling & Biju, 2018	Mahony et al. 2018
*M. oropedion* Mahony, Teeling & Biju, 2013	Mahony et al. 2013
*M. orientalis* Li, Lyu, Wang & Wang, 2020	Li et al. 2020
*M. pachyproctus* Huang, 1981	Fei et al. 2009
*M. palpebralespinosa* Bourret, 1937	Fei et al. 2012
*M. parallela* Inger & Iskandar, 2005	Inger and Iskandar 2005
*M. parva* (Boulenger, 1893)	Fei et al. 2009
*M. periosa* Mahony, Kamei, Teeling & Biju, 2018	Mahony et al. 2018
*M. popei* (Zhao, Yang, Chen, Chen & Wang, 2014)	Zhao et al. 2014
*M. robusta* Boulenger, 1908	Mahony et al. 2018
*M. rubrimera* Tapley, Cutajar, Mahony, Chung, Dau, Nguyen, Luong & Rowley, 2017	Tapley et al. 2017
*M. sangzhiensis* Jiang, Ye & Fei, 2008	Jiang et al. 2008
*M. serchhipii* (Mathew & Sen, 2007)	Mathew and Sen 2007
*M. shapingensis* Liu, 1950	Fei et al. 2009
*M. shuichengensis* Tian & Sun, 1995	Fei et al. 2009
*M. shunhuangensis* Wang, Deng, Liu, Wu & Liu, 2019	Wang et al. 2019a
*M. spinata* Liu & Hu, 1973	Fei et al. 2009
*M. stejnegeri* Taylor, 1920	Taylor 1920
*M. synoria* (Stuart, Sok & Neang, 2006)	Stuart et al. 2006
*M. takensis* Mahony, 2011	Mahony 2011
*M. tuberogranulata* Shen, Mo & Li, 2010	Fei et al. 2012
*M. vegrandis* Mahony, Teeling & Biju, 2013	Mahony et al. 2013
*M. wawuensis* Fei, Jiang & Zheng, 2001	Fei et al. 2012
*M. wugongensis* Wang, Lyu & Wang, 2019	Wang et al. 2019b
*M. wuliangshanensis* Ye & Fei, 1995	Fei et al. 2012
*M. wushanensis* Ye & Fei, 1995	Fei et al. 2012
*M. xianjuensis* Wang, Wu, Peng, Shi, Lu & Wu, 2020	Wang et al. 2020
*M. zhangi* Ye & Fei, 1992	Fei et al. 2012
*M. zunhebotoensis* (Mathew & Sen, 2007)	Mathew and Sen 2007

### Bioacoustics notes

Ten advertisement calls from two individuals of the new species were recorded on 18 May 2018 between 21:00–23:00 in Chishui City, Guizhou Province, China in the field. SONY PCM-D50 digital sound recorder was used to record within 20 cm of the calling individuals. The sound files in wave format were resampled at 48 kHz with sampling depth 24 bits. The sonograms and waveforms were generated by WaveSurfer software (Sjöander and Beskow 2000) from which all parameters and characters were measured. Ambient temperature was taken by a digital hygrothermograph.

## Results

### Phylogenetic analyses

**Figure 2.**
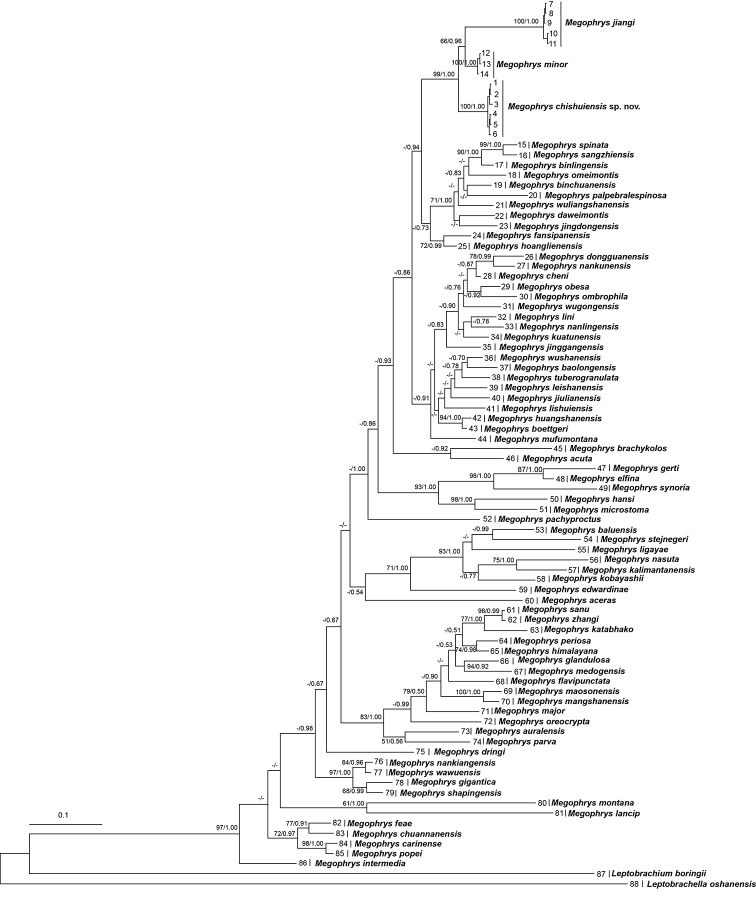
Maximum likelihood (ML) tree of the genus *Megophrys* reconstructed based on the 16S rRNA and COI gene sequences. Bayesian posterior probability/ML bootstrap supports were denoted beside each node. Samples 1–88 refer to Suppl. material 2: Table S2.

**Figure 3.**
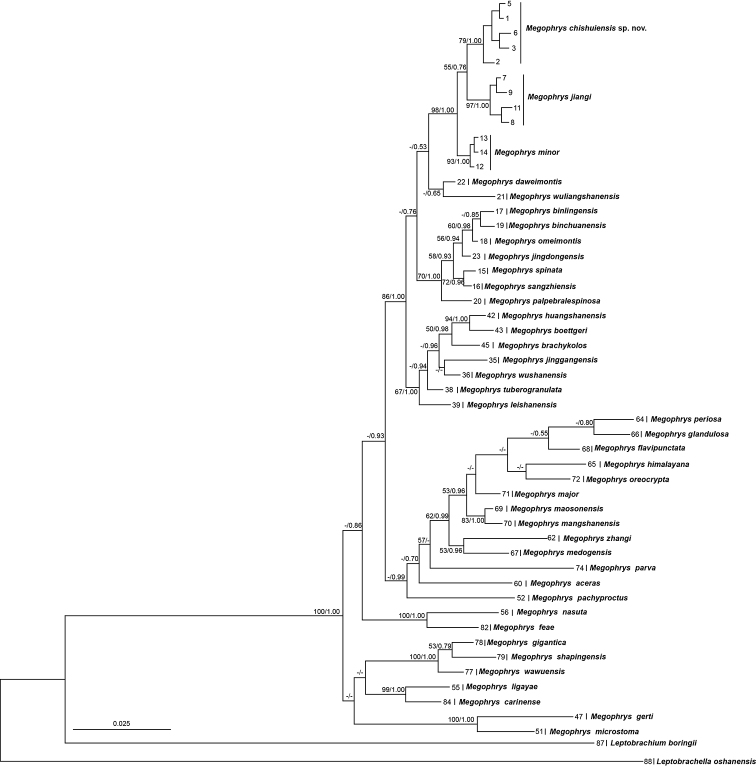
Maximum likelihood (ML) tree of the genus *Megophrys* reconstructed based on the nuclear DNA sequences of RAG1 and BDNF genes. Bayesian posterior probability/ML bootstrap supports were denoted beside each node. Samples 1–88 refer to Suppl. msterial 2: Table S2.

Aligned sequence matrix of 16S+COI and RAG1+BDNF contains 1104 bp and 1582 bp, respectively. ML and BI trees of the mitochondrial DNA dataset presented almost consistent topology (Fig. 2), and as well, ML and BI trees of the nuclear DNA dataset showed almost identical topology (Fig. 3), though relationships of many lineages were unresolved (Figs 2, 3). In mitochondrial DNA trees, the undescribed species was clustered as an independent clade sister to a clade in comprising of *M. minor* Stejneger, 1926 and *M. jiangi* Liu, Li, Wei, Xu, Cheng, Wang & Wu, 2020, but in nuclear DNA trees, the undescribed species clade was sister to *M. jiangi*, and then was clustered together with *M. minor*.

Genetic distances on16S gene with uncorrected *p*-distance model between samples of the undescribed species were below 0.2%. The genetic distance between the undescribed species and its closest related species *M. minor* was 2.2% on 16S gene, which was higher or at the same level with those among many pairs of congeners, for example, 1.7% between *M. spinata* Liu & Hu, 1973 and *M. sangzhiensis* Jiang, Ye & Fei, 2008, 2.1% between *M. omeimontis* Liu, 1950 and *M. binlingensis* Jiang, Fei & Ye, 2009, and 2.2% between *M. cheni* (Wang & Liu, 2014) and *M. nankunensis* Wang, Zeng & Wang, 2019; Suppl. material 4: Table S4).

## Taxonomic accounts

### Megophrys chishuiensis sp. nov.

Taxon classificationAnimaliaAnuraMegophryidae

6C144FD5-3754-53EB-A648-CF06D30DC197

http://zoobank.org/20B6A80B-E937-4443-88A2-E357B77DB6CA

**Figure 4.**
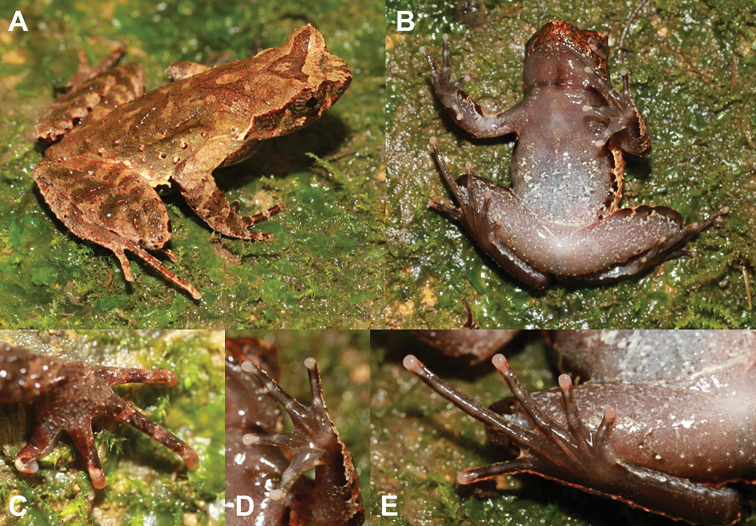
Photos of the holotype CIBCS20190518031 of *Megophrys chishuiensis* sp. nov. in life **A** dorsal view **B** ventral view **C** dorsal view of hand **D** ventral view of hand **E** ventral view of foot.

**Figure 5.**
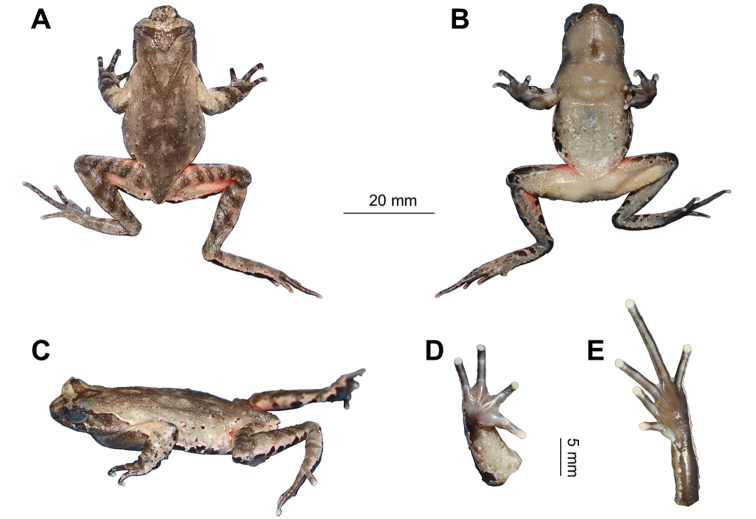
The holotype specimen CIBCS20190518031 of *Megophrys chishuiensis* sp. nov. **A** dorsal view **B** ventral view **C** lateral view **D** ventral view of hand **E** ventral view of foot.

**Figure 6.**
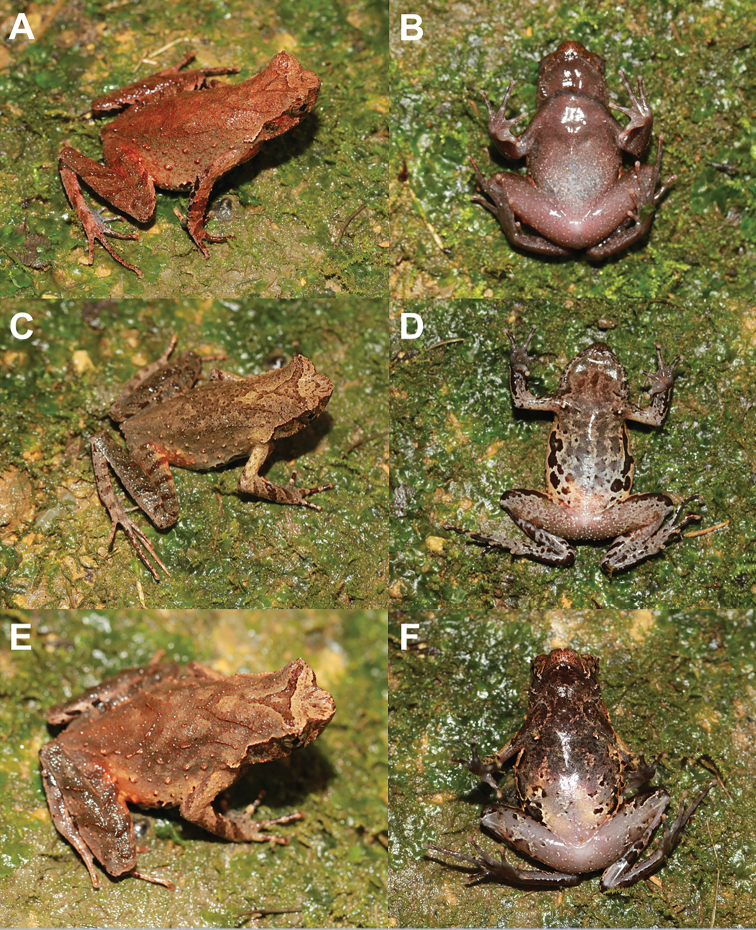
Color variation in *Megophrys chishuiensis* sp. nov. **A** dorsolateral view of the female specimen CIBCS20190518027 **B** dorsolateral view of the female specimen CIBCS20190518030 **C** dorsal view of the female specimen CIBCS20190518025 **D** ventral view of the male specimen CIBCS20190518019 **E** ventral view of the female specimen CIBCS20190518030 **F** ventral view of the female specimen CIBCS20190518025.

**Figure 7.**
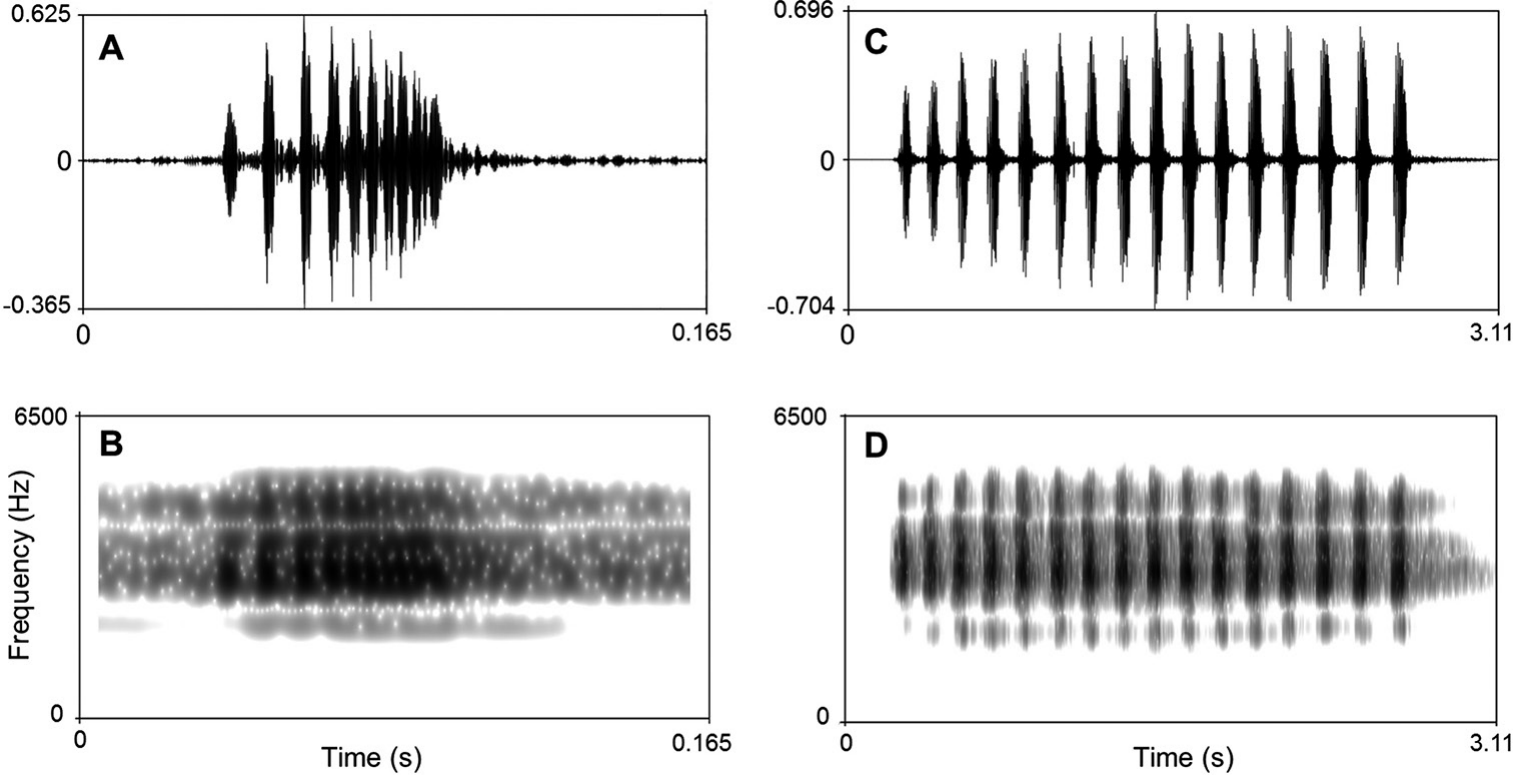
Visualization of advertisement calls of *Megophrys chishuiensis* sp. nov. **A** waveform showing one note **B** sonogram showing one notes **C** waveform showing 16 notes of one call **D** sonogram showing 16 notes of one call.

**Figure 8.**
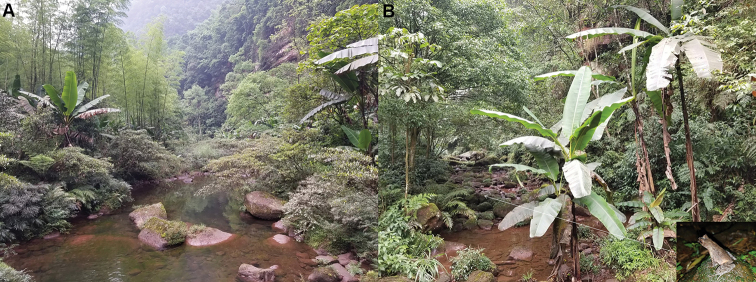
Habitats of *Megophrys chishuiensis* sp. nov. in the type locality, Chishui National Nature Reserve, Chishui City, Guizhou Province, China **A** landscape of montane forests **B** a mountain stream (the inset illustrates the holotype on stone).

Figures 4 , 5 , 6 , 7 , 8

#### Type material.

***Holotype*.** CIBCS20190518031 (Figs 4, 5), adult male, from Chishui National Nature Reserve, Chishui City, Guizhou Province, China (28.436708N, 105.997794E, ca. 460 m a. s. l.), collected by Shi-Ze Li on 18 May 2019.

***Paratype*.** Two adult males and five adult females from the same place as holotype, collected by Shi-Ze Li and Jing Liu. Two females CIBCS20190518022 and CIBCS20190518023 collected by Jing LIU on 18 May 2019, two adult males CIBCS20190518019 and CIBCS20190518021 and three adult females CIBCS20190518025, CIBCS20190518027 and CIBCS20190518030 collected by Shi-Ze Li on 18 May 2019.

#### Diagnosis.

*Megophrys chishuiensis* sp. nov. is assigned to the genus *Megophrys* based on molecular phylogenetic analyses and the following generic diagnostic characters: snout shield-like; projecting beyond the lower jaw; canthus rostralis distinct; chest glands small and round, closer to the axilla than to midventral line; femoral glands on rear part of thigh; vertical pupils (Fei et al. 2009).

*Megophrys chishuiensis* sp. nov. could be distinguished from its congeners by a combination of the following morphological characters: (1) body size moderate (SVL 43.4–44.1 mm in males, and 44.8–49.8 mm in females; (2) vomerine teeth absent; (3) tongue not notched behind; (4) a small horn-like tubercle at the edge of each upper eyelid; (5) tympanum distinctly visible, rounded; (6) two metacarpal tubercles on palm; (7) relative finger lengths II < I < V < III; (8) toes without webbing; (9) heels overlapping when thighs are positioned at right angles to the body; (10) tibiotarsal articulation reaching the level between tympanum and eye when leg stretched forward. In breeding male, (11) an internal single subgular vocal sac; (12) nuptial pads with black spines on dorsal surface of bases of the first two fingers.

#### Description of holotype.

(Figs 4, 5). SVL 43.4 mm; head width larger than head length (HDW/HDL ratio about 1.2); snout obtusely pointed, protruding well beyond the margin of the lower jaw in ventral view; loreal region vertical and concave; canthus rostralis well-developed; top of head flat in dorsal view; a small horn-like tubercle at the edge of the upper eyelid; eye large, eye diameter 43.9% of head length; pupils vertical; nostril orientated laterally, closer to snout than eye; tympanum distinct, TYP/EYE ratio 0.64; vomerine ridges and vomerine teeth absent; margin of tongue smooth, not notched behind.

Forelimbs slender, the length of lower arm and hand 42.4% of SVL; fingers slender, relative finger lengths: II < I < V < III; tips of digits globular, without lateral fringes; subarticular tubercle distinct at the base of each finger; two metacarpal tubercles, prominent, the outer one long and thin, the inner one oval-shaped.

Hindlimbs slender, 1.48 times SVL; heels overlapping when thighs are positioned at right angles to the body, tibiotarsal articulation reaching tympanum to eye when leg stretched forward; tibia length longer than thigh length; relative toe lengths I < II < V < III < IV; tips of toes round, slightly dilated; subarticular tubercles absent; toes without webbing; no lateral fringe; inner metatarsal tubercle oval-shaped; outer metatarsal tubercle absent.

Dorsal skin rough, with numerous granules; several large warts scattered on flanks; a small horn-like tubercle at the edge of each upper eyelid; tubercles on the dorsum forming a weak X-shaped ridge, the V-shaped ridges disconnect; two discontinuous dorsolateral parallel ridges on either side of the X-shaped ridges; an inverted triangular brown speckle between two upper eyelids; several tubercles on the flanks and dorsal surface of thighs and tibias and forming four transverse tubercle rows; supratympanic fold distinct.

Ventral surface smooth; chest with small and round glands, closer to the axilla than to midventral line; femoral glands on rear of thighs, numerous white granules on outer thighs; posterior end of the body distinctly protruding and forming an arc-shaped swelling above the anal region.

#### Coloration of holotype in life.

(Fig. 4). An inverted triangular brown speckle between the eyes; X-shaped ridges on the dorsum, four transverse bands on the dorsal surface of the thigh and shank; several dark brown and white vertical bars on the lower and upper lip; venter purple grey, some white spots on the ventral surface of body and limbs; palms and soles uniform purple grey, tip of digits pinkish; pectoral and femoral glands white.

#### Coloration of holotype in preservation.

(Fig. 5). Color of dorsal surface fades to olive; the inverted triangular brown speckle between the eyes, X-shaped ridges on dorsum and transverse bands on limbs and digits distinct; ventral surface greyish white; creamy-white substitutes the pinkish on tip of digits; the posterior of ventral surface of body, inner of thigh and upper of tibia light red.

#### Variations.

In CIBCS20190518027, the back is brown with some brick-red granules (Fig. 6A); in CIBCS20190518030, the X-shaped marking on back of trunk consists of a ridge with brown spots (Fig. 6B), and the throat and anterior belly are purplish, with grey spots on the posterior belly and black spots on the flank belly (Fig. 6E); in CIBCS20190518025, the marking on the back consists of a V-shaped ridge (Fig. 6C), and the anterior belly is brownish with some black spots on flank and belly, and posterior belly is beige (Fig. 6F); in CIBCS20190518019, the whole ventrum is purplish except the posterior belly that shows white blotches (Fig. 6D).

#### Advertisement call.

The call description is based on recordings of the holotype CIBCS20190518031 (Fig. 7) from the shrub leaf near the streamlet, and the ambient air temperature was 24.5 °C. Each call consists of 14–20 (mean 16.14 ± 1.95, *N* = 10) notes. Call duration was 2.10–3.18 second (mean 2.51 ± 0.33, *N* = 7). Call interval was 0.92–1.32 seconds (mean 1.13 ± 0.15, *N* = 6). Each note had a duration of 0.07– 0.12 seconds (mean 0.98 ± 0.01, *N* = 113) and the intervals between notes 0.038–0.085 seconds (mean 0.056 ± 0.011, *N* = 106). Amplitude modulation within note was apparent, beginning with moderately high energy pulses, increasing slightly to a maximum by approximately mid note, and then decreasing towards the end of each note. The average dominant frequency was 5859 ± 118.02.61 (5733–6064 Hz, *N* = 7).

#### Secondary sexual characters.

Adult females with SVL 44.8–49.8 mm, larger than adult males with 43.4–44.1 mm. Adult males have a single subgular vocal sac. In breeding males, brownish red nuptial pads are present on dorsal surface of the bases of the first and second fingers with black spines obvious under microscope.

#### Comparisons.

By having medium body size, *Megophrys chishuiensis* sp. nov. differs from *M. aceras* Boulenger, 1903, *M. auralensis* Ohler, Swan & Daltry, 2002, *M. carinense* Boulenger, 1889, *M. caudoprocta* Shen, 1994, *M. chuannanensis* (Fei, Ye & Huang, 2001), *M. damrei* Mahony, 2011, *M. edwardinae* Inger, 1989, *M. feae* Boulenger, 1887, *M. flavipunctata* Mahony, Kamei, Teeling & Biju, 2018, *M. gigantica* Liu, Hu & Yang, 1960, *M. glandulosa* Fei, Ye & Huang, 1990, *M. himalayana* Mahony, Kamei, Teeling & Biju, 2018, *M. intermedia* Smith, 1921, *M. jingdongensis* Fei & Ye, 1983, *M. kalimantanensis* Munir, Hamidy, Matsui, Iskandar, Sidik & Shimada, 2019, *M. lekaguli* Stuart, Chuaynkern, Chan-ard & Inger, 2006, *M. liboensis* (Zhang, Li, Xiao, Li, Pan, Wang, Zhang & Zhou, 2017), *M. major* Boulenger, 1908, *M. mangshanensis* Fei & Ye, 1990, *M. maosonensis* Bourret, 1937, *M. medogensis* Fei, Ye & Huang, 1983, *M. omeimontis* Liu, 1950, *M. oreocrypta* Mahony, Kamei, Teeling & Biju, 2018, *M. orientalis* (Li, Lyu, Wang & Wang, 2020), *M. periosa* Mahony, Kamei, Teeling & Biju, 2018, *M. popei* (Zhao, Yang, Chen, Chen & Wang, 2014), *M. sangzhiensis* Jiang, Ye & Fei, 2008, *M. shapingensis* Liu, 1950, *M. shuichengensis* Tian & Sun, 1995, and *M. takensis* Mahony, 2011 (maximum SVL < 49.8 mm in the new species vs. minimum SVL > 53 mm in the latter), and differs from *M. acuta* Wang, Li & Jin, 2014, *M. angka* (Wu, Suwannapoom, Poyarkov, Chen, Pawangkhanant, Xu, Jin, Murphy & Che, 2019), *M. caobangensis* Nguyen, Pham, Nguyen, Luong & Ziegler, 2020, *M. damrei* Mahony, 2011, *M. dongguanensis* Wang & Wang, 2019, *M. cheni*, *M. jiangi*, *M. jinggangensis* (Wang, 2012), *M. jiulianensis* Wang, Zeng, Lyu & Wang, 2019, *M. kuatunensis* Pope, 1929, *M. lini* (Wang & Yang, 2014), *M. lishuiensis* (Wang, Liu & Jiang, 2017), *M. mufumontana* (Wang, Lyu & Wang, 2019), *M. minor*, *M. nanlingensis* (Lyu, Wang, Liu & Wang, 2019), *M. obesa* Wang, Li & Zhao, 2014, *M. pachyproctus* Huang, 1981, *M. palpebralespinosa* Bourret, 1937, *M. serchhipii* Mathew & Sen, 2007, *M. shunhuangensis* Wang, Deng, Liu, Wu & Liu, 2019, *M. vegrandis* Mahony, Teeling & Biju, 2013, *M. wuliangshanensis* Ye & Fei, 1995, *M. wushanensis* Ye & Fei, 1995, *M. zunhebotoensis* Mathew & Sen, 2007, *M. xianjuensis* Wang, Wu, Peng, Shi, Lu & Wu, 2020, and *M. zhangi* Ye & Fei, 1992 (vs. maximum SVL < 42 mm in the latter).

By the absence of vomerine teeth, *Megophrys chishuiensis* sp. nov. differs from *M. aceras*, *M. ancrae* Mahony, Teeling & Biju, 2013, *M. carinense*, *M. baluensis* (Boulenger, 1899), *M. caudoprocta*, *M. chuannanensis*, *M. damrei*, *M. daweimontis* Rao & Yang, 1997, *M. dongguanensis*, *M. fansipanensis* Tapley, Cutajar, Mahony, Nguyen, Dau, Luong, Le, Nguyen, Nguyen, Portway, Luong & Rowley, 2018, *M. flavipunctata*, *M. glandulosa*, *M. hoanglienensis* Tapley, Cutajar, Mahony, Nguyen, Dau, Luong, Le, Nguyen, Nguyen, Portway, Luong & Rowley, 2018, *M. himalayana*, *M. insularis* (Wang, Liu, Lyu, Zeng & Wang, 2017), *M. intermedia*, *M. jingdongensis*, *M. jinggangensis*, *M. jiulianensis*. *M. kalimantanensis*, *M. kobayashii* Malkmus & Matsui, 1997, *M. lancip* Munir, Hamidy, Farajallah & Smith, 2018, *M. lekaguli*, *M. liboensis*, *M. ligayae* Taylor, 1920, *M. longipes* Boulenger, 1886, *M. major*, *M. mangshanensis*, *M. maosonensis*, *M. medogensis*, *M. megacephala* Mahony, Sengupta, Kamei & Biju, 2011, *M. montana* Kuhl & Van Hasselt, 1822, *M. nasuta* (Schlegel, 1858), *M. nankunensis*, *M. nanlingensis*, *M. omeimontis*, *M. oropedion* Mahony, Teeling & Biju, 2013, *M. oreocrypta*, *M. palpebralespinosa*, *M. parallela* Inger & Iskandar, 2005, *M. parva* (Boulenger, 1893), *M. periosa*, *M. popei*, *M. robusta* Boulenger, 1908, *M. rubrimera* Tapley, Cutajar, Mahony, Chung, Dau, Nguyen, Luong & Rowley, 2017, *M. sangzhiensis*, *M. stejnegeri* Taylor, 1920, *M. takensis*, *M. zhangi*, and *M. zunhebotoensis* (vs. present in the latter).

By having a small horn-like tubercle at the edge of each upper eyelid, *Megophrys chishuiensis* sp. nov. differs from *M. binchuanensis* Ye & Fei, 1995, *M. binlingensis*, *M. damrei*, *M. gigantica*, *M. minor*, *M. monticola* (Günther, 1864), *M. nasuta*, *M. nankiangensis* Liu & Hu, 1966, *M. oropedion*, *M. pachyproctus*, *M. spinata*, *M. stejnegeri*, *M. takensis*, *M. wuliangshanensis*, *M. wushanensis*, *M. zhangi*, and *M. zunhebotoensis* (vs. lacking tubercle in the latter), and differs from *M. carinense*, *M. feae*, *M. gerti* (Ohler, 2003), *M. hansi* (Ohler, 2003), *M. intermedia*, *M. kalimantanensis*, *M. koui* Mahony, Foley, Biju & Teeling, 2017, *M. latidactyla*, *M. liboensis*, *M. microstoma* (Boulenger, 1903), *M. palpebralespinosa*, *M. popei*, *M. shuichengensis*, and *M. synoria* (Stuart, Sok & Neang, 2006) (vs. having a prominent and elongated tubercle in the latter).

By having a tongue not notched behind, *Megophrys chishuiensis* sp. nov. differs from *M. ancrae*, *M. baolongensis* Ye, Fei & Xie, 2007, *M. binlingensis*, *M. boettgeri* (Boulenger, 1899), *M. carinense*, *M. cheni*, *M. chuannanensis*, *M. damrei*, *M. dringi* Inger, Stuebing & Tan, 1995, *M. fansipanensis*, *M. feae*, *M. feii* Yang, Wang & Wang, 2018, *M. flavipunctata*, *M. gerti*, *M. glandulosa*, *M. hoanglienensis*, *M. huangshanensis* Fei & Ye, 2005, *M. insularis*, *M. jiulianensis*. *M. jingdongensis*, *M. kalimantanensis*, *M. kuatunensis*, *M. liboensis*, *M. mangshanensis*, *M. maosonensis*, *M. medogensis*, *M. minor*, *M. nankiangensis*, *M. nanlingensis*, *M. omeimontis*, *M. oropedion*, *M. pachyproctus*, *M. parallela*, *M. popei*, *M. robusta*, *M. sangzhiensis*, *M. shapingensis*, *M. shuichengensis*, *M. spinata*, *M. vegrandis*, *M. wawuensis* Fei, Jiang & Zheng, 2001, *M. zhangi*, and *M. zunhebotoensis* (vs. tongue notched behind in the latter).

By lacking lateral fringes on the toes, *Megophrys chishuiensis* sp. nov. differs from *M. acuta*, *M. auralensis*, *M. baolongensis*, *M. binchuanensis*, *M. boettgeri*, *M. carinense*, *M. cheni*, *M. chuannanensis*, *M. elfina* Poyarkov, Duong, Orlov, Gogoleva, Vassilieva, Nguyen, Nguyen, Nguyen, Che & Mahony, 2017, *M. feae*, *M. feii*, *M. flavipunctata*, *M. gigantica*, *M. glandulosa*, *M. hansi*, *M. intermedia*, *M. jingdongensis*, *M. jinggangensis*, *M. kuatunensis*, *M. latidactyla*, *M. lini*, *M. major*, *M. maosonensis*, *M. nankiangensis*, *M. omeimontis*, *M. palpebralespinosa*, *M. popei*, *M. rubrimera*, *M. sangzhiensis*, *M. serchhipii*, *M. shapingensis*, *M. shuichengensis*, *M. spinata*, *M. vegrandis*, *M. xianjuensis*, *M. zhangi*, and *M. zunhebotoensis* (vs. present in these species).

By having toes without webs at bases, *Megophrys chishuiensis* sp. nov. differs from *M. brachykolos* Inger & Romer, 1961, *M. carinense*, *M. flavipunctata*, *M. jingdongensis*, *M. jinggangensis*, *M. lini*, *M. major*, *M. palpebralespinosa*, *M. popei*, *M. shuichengensis*, *M. spinata* (vs. at least one-fourth webbed).

By heels overlapping when thighs are positioned at right angles to the body, *Megophrys chishuiensis* sp. nov. differs from *M. acuta*, *M. brachykolos*, *M. dongguanensis*, *M. huangshanensis*, *M. kuatunensis*, *M. nankunensis*, *M. obesa*, *M. ombrophila* Messenger & Dahn, 2019, and *M. wugongensis* Wang, Lyu & Wang, 2019 (vs. not meeting).

With tibiotarsal articulation reaching to the level between tympanum and eye when leg is stretched forward, *Megophrys chishuiensis* sp. nov. differs from *M. baolongensis*, *M. nankiangensis*, *M. pachyproctus*, *M. shuichengensis* and *M. tuberogranulata* Shen, Mo & Li, 2010 (vs. just reaching posterior corner of the eye in the latter); differs from *M. daweimontis*, *M. glandulosa*, *M. lini*, *M. major*, *M. medongensis*, *M. obesa*, and *M. sangzhiensis* (vs. reaching the anterior corner of the eye or beyond eye or nostril and tip of snout in the latter); differs from *M. leishanensis* Li, Xu, Liu, Jiang, Wei & Wang, 2018 (vs. reaching middle part of eye in this group of species); and differs from *M. mufumontana* (vs. reaching tympanum in males and to the eye in females).

By having an internal single subgular vocal sac in male, *Megophrys chishuiensis* sp. nov. differs from *M. caudoprocta*, *M. shapingensis*, and *M. shuichengensis* (vs. vocal sac absent).

By having nuptial pads and nuptial spines on dorsal surface of the base of the first two fingers in breeding males, *Megophrys chishuiensis* sp. nov. differs from *M. acuta*, *M. feii*, *M. shapingensis*, and *M. shuichengensis* (vs. lacking in these species).

The congeners *M. carinense*, *M. jiangi*, *M. leishanensis*, *M. liboensis*, *M. shuichengensis*, and *M. spinata* have sympatric distribution with *Megophrys chishuiensis* sp. nov. (Fei et al. 2012; Zhang et al. 2017; Li et al. 2018; Liu et al. 2020) . The new species can be distinguished from these species by a series of morphological characters as follows. The new species vs. *M. carinense*: body size smaller (adult males with 43.4–44.1 mm and adult females with SVL 44.8–49.8 mm vs. adult males with 92–123 mm and adult females with SVL 137mm), vomerine teeth absent (vs. present), horn-like tubercle at the edge of each upper eyelid small (vs. prominent), tongue not notched behind (vs. notched behind), lacking lateral fringe in toes (vs. present), and toes without webs at bases (vs. one-fourth webbed). The new species vs. *M. jiangi*: body size bigger (adult males with 43.4–44.1 mm and adult females with SVL 44.8–49.8 mm vs. adult males with 34.4–39.2 mm and adult females with SVL 39.5–40.4 mm), and relative finger lengths II < I < V < III vs. I < II < V < III. The new species vs. *M. leishanensis*: body size bigger (adult males with 43.4–44.1 mm and adult females with SVL 44.8–49.8 mm vs. adult males with 30.4–38.7 mm and adult females with SVL 42.3 mm), and tibiotarsal articulation reaching forward to the region between tympanum and eye when hindlimb is stretched along the side of the body vs. reaching middle part of eye. The new species vs. *M. liboensis*: body size smaller in adult females (adult females with SVL 44.8–49.8 mm vs. adult females with SVL 60.8–70.6 mm), vomerine teeth absent vs. vomerine teeth present, and horn-like tubercle at the edge of each upper eyelid is small vs. prominent. The new species vs. *M. shuichengensis*: body size smaller (adult males with 43.4–44.1 mm and adult females with SVL 44.8–49.8 mm vs. adult males with 102.0–118.3 mm and adult females with SVL 99.8–115.6 mm), horn-like tubercle at the edge of each upper eyelid is small vs. prominent, tongue not notched behind vs. tongue notched behind, lacking lateral fringe in toes vs. present, toes without webs at bases vs. one-fourth webbed, having an internal single subgular vocal sac in male vs. absent, and having nuptial pads and nuptial spines on the dorsal base of the first two fingers in breeding male vs. lacking. The new species vs. *M. spinata*: body size is smaller (adult males with 43.4–44.1 mm and adult females with SVL 44.8–49.8 mm vs. adult males with 47.2–54.4 mm and adult females with SVL 54.0–55.0 mm), horn-like tubercle at the edge of each upper eyelid is small vs. lacking tubercle, tongue not notched behind vs. notched behind, lacking lateral fringe in toes vs. present, and toes without webs at bases vs. one-fourth webbed.

*Megophrys chishuiensis* sp. nov. is phylogenetically closest to *M. minor*, and this new species could be identified from the latter distinctly by having larger body size (SVL 43.4–44.1 mm in males vs. 34.5–41.2 mm in males of *M. minor*), having a small horn-like tubercle at the edge of each upper eyelid (vs. absent in the latter), tongue not notched behind (vs. notched in the latter), tibiotarsal articulation reaching the level between tympanum to eye when leg stretched forward (vs. reaching the level between eye and tip of snout in the latter), and having two metatarsal tubercles in each hand (vs. absent in the latter).

#### Distribution and habitats.

*Megophrys chishuiensis* sp. nov. is known from the type locality, Chishui National Nature Reserve (28.38–28.45N, 106.05–109.75E), Chishui City, Guizhou Province, China at elevations between 270–604 m. The individuals of the new species were frequently found in bamboo forest nearby the streams (Fig. 8), and five sympatric amphibian species were also found: *Megophrys omeimontis*, *Odorrana margaratae* (Liu, 1950), *Zhangixalus omeimontis* (Stejneger, 1924), and *Rana omeimontis* Ye & Fei, 1993.

#### Etymology.

The specific name *chishuiensis* refers to the distribution of this species, Chishui City, Guizhou Province, China. We propose the common name “Chishui horned toad” and its Chinese name as Chi Shui Jiao Chan (赤水角蟾).

## Discussion

The new species, *Megophrys chishuiensis* sp. nov., resembles *M. minor* and *M. jiangi*, and detailed comparison with different data sets are important for recognizing them. Our molecular phylogenetic data on mitochondrial DNA and nuclear DNA, and morphological comparisons both separated the new species from the two closely related species. *Megophrys minor* were reported to be distributed widely through the provinces of Sichuan, Guizhou, Chongqing, Yunnan, Guangxi, Jiangxi and north of Vietnam (Fei et al. 2012), but detailed investigations with multiple data suggested that several populations of the species should contain cryptic species (including *Megophrys chishuiensis* sp. nov. and *M. jiangi*). In recent years, a lot of new species of the genus *Megophrys* have been gradually described, of which, a large part of number of species were found in China (Frost 2020). To now, among the 97 species of *Megophrys*, 51 species were discovered in China. Even so, dozens of cryptic species need to be described (Chen et al. 2016; Liu et al. 2018) just in China. Obviously, we should conduct more investigations on the differentiation of the populations and explore the species identity in the wide range.

*Megophrys chishuiensis* sp. nov. with a narrow distribution also fits the “micro-endemism” model like many other congeners (Liu et al. 2018; Wang et al. 2019). Besides, the new species is likely to be threatened by several factors, i.e., developing tourism in Chishui National Nature Reserve, constructions in this area and increasing pollution from tourists. Reasonable managements of tourism in this area may probably facilitate the protection of the populations of the toad and other animal species.

## Supplementary Material

XML Treatment for Megophrys chishuiensis

## Supplementary materials

Supplementary material 1 Table S1 Explanation note Measurements of the adult specimens of *Megophrys chishuiensis* sp. nov. https://binary.pensoft.net/file/424145This dataset is made available under the Open Database License (http://opendatacommons.org/licenses/odbl/1.0/). The Open Database License (ODbL) is a license agreement intended to allow users to freely share, modify, and use this Dataset while maintaining this same freedom for others, provided that the original source and author(s) are credited.Ning Xu, Shi-Ze Li, Jing Liu, Gang Wei, Bin Wang

Supplementary material 2 Table S2 Explanation note Localities, voucher information, and GenBank accession numbers for molecular samples used in this study. https://binary.pensoft.net/file/424146This dataset is made available under the Open Database License (http://opendatacommons.org/licenses/odbl/1.0/). The Open Database License (ODbL) is a license agreement intended to allow users to freely share, modify, and use this Dataset while maintaining this same freedom for others, provided that the original source and author(s) are credited.Ning Xu, Shi-Ze Li, Jing Liu, Gang Wei, Bin Wang

Supplementary material 3 Table S3 Explanation note Primer sequences used in this study. https://binary.pensoft.net/file/424147This dataset is made available under the Open Database License (http://opendatacommons.org/licenses/odbl/1.0/). The Open Database License (ODbL) is a license agreement intended to allow users to freely share, modify, and use this Dataset while maintaining this same freedom for others, provided that the original source and author(s) are credited.Ning Xu, Shi-Ze Li, Jing Liu, Gang Wei, Bin Wang

Supplementary material 4 Table S4 Explanation note Uncorrected p-distances between the *Megophrys* species based on the 16S gene sequences. https://binary.pensoft.net/file/424148This dataset is made available under the Open Database License (http://opendatacommons.org/licenses/odbl/1.0/). The Open Database License (ODbL) is a license agreement intended to allow users to freely share, modify, and use this Dataset while maintaining this same freedom for others, provided that the original source and author(s) are credited.Ning Xu, Shi-Ze Li, Jing Liu, Gang Wei, Bin Wang
