# Supplementary material for: A new species of the horned toad Megophrys Kuhl & Van Hasselt, 1822 (Anura, Megophryidae) from southwest China
Source: Zookeys. 2020 Jun 22;943:119–44. doi: 10.3897/zookeys.943.50343 (PMC7326726; doi:10.3897/zookeys.943.50343)
Supplement: Supplementary material 3 — Table S3 [file zookeys-943-119-s003.doc]

**Table S3.** Primer sequences used in this study.

| Locus | Primer name | Sequence (5′–3′) | Source |
| --- | --- | --- | --- |
| 16S rRNA | P7 | CGCCTGTTTACCAAAAACAT | Simon et al. 1994 |
|  | P8 | CCGGTCTGAACTCAGATCACGT |  |
| COI | Chmf4 | TYTCWACWAAYCAYAAAGAYATCGG | Che et al. 2012 |
|  | Chmr4 | ACYTCRGGRTGRCCRAARAATCA |  |
| RAG1 | Rag1_1F | GCMTTGCTSCCRGGGTATCA | Shen et al*.* 2013 |
|  | Rag1_2R | TCAATGGACGGAAGGGTTTCAATAA |  |
| BDNF | BDNF-F | ACCATCCTTTTCCTKACTATGG | Vieites et al. 2007 |
|  | BDNF-R | CTATCTTCCCCTTTTAATGGTC |  |
